# ChatGPT in Medical Education: Bibliometric and Visual Analysis

**DOI:** 10.2196/72356

**Published:** 2025-10-07

**Authors:** Yuning Zhang, Xiaolu Xie, Qi Xu

**Affiliations:** 1 School of Basic Medical Sciences, Gannan Medical University, Ganzhou, China; 2 School of Medical and Information Engineering, Gannan Medical University, Ganzhou, China; 3 School of Public Health and Health Management, Gannan Medical University, 1 Harmony Avenue, Rongjiang New District, Ganzhou, 341000, China, 86 15770701553

**Keywords:** ChatGPT, medical education, bibliometric, VOSviewer, CiteSpace, artificial intelligence, AI

## Abstract

**Background:**

ChatGPT is a generative artificial intelligence–based chatbot developed by OpenAI. Since its release in the second half of 2022, it has been widely applied across various fields. In particular, the application of ChatGPT in medical education has become a significant trend. To gain a comprehensive understanding of the research developments and trends regarding ChatGPT in medical education, we conducted an extensive review and analysis of the current state of research in this field.

**Objective:**

This study used bibliometric and visualization analysis to explore the current state of research and development trends regarding ChatGPT in medical education.

**Methods:**

A bibliometric analysis of 407 articles on ChatGPT in medical education published between March 2023 and June 2025 was conducted using CiteSpace, VOSviewer, and Bibliometrix (RTool of RStudio). Visualization of countries, institutions, journals, authors, keywords, and references was also conducted.

**Results:**

This bibliometric analysis included a total of 407 studies. Research in this field began in 2023, showing a notable surge in annual publications until June 2025. The United States, China, Türkiye, the United Kingdom, and Canada produced the most publications. Networks of collaboration also formed among institutions. The University of California system was a core research institution, with 3.4% (14/407) of the publications and 0.17 betweenness centrality. *BMC Medical Education*, *Medical Teacher*, and the *Journal of Medical Internet Research* were all among the top 10 journals in terms of both publication volume and citation frequency. The most prolific author was Yavuz Selim Kiyak, who has established a stable collaboration network with Isil Irem Budakoglu and Ozlem Coskun. Author collaboration in this field is usually limited, with most academic research conducted by independent teams and little communication between teams. The most frequent keywords were “AI,” “ChatGPT,” and “medical education.” Keyword analysis further revealed “educational assessment,” “exam,” and “clinical practice” as current research hot spots. The most cited paper was “Performance of ChatGPT on USMLE: Potential for AI-Assisted Medical Education Using Large Language Models,” and the paper with the strongest citation burst was “Are ChatGPT’s Knowledge and Interpretation Ability Comparable to Those of Medical Students in Korea for Taking a Parasitology Examination?: A Descriptive Study.” Both papers focus on evaluating ChatGPT’s performance in medical exams.

**Conclusions:**

This study reveals the significant potential of ChatGPT in medical education. As the technology improves, its applications will expand into more fields. To promote the diversification and effectiveness of ChatGPT in medical education, future research should strengthen interregional collaboration and enhance research quality. These findings provide valuable insights for researchers to identify research perspectives and guide future research directions.

## Introduction

### Background

Large language models (LLMs) represent major progress in artificial intelligence (AI), especially for computational linguistics and natural language processing. These generative AI models are fundamentally based on the transformer neural network architecture [1]. Training is conducted using extensive text datasets, including books, documents, and website content. LLMs have been developed to predict subsequent words or tokens. Through this process, they learn to recognize complex language patterns, including vocabulary, grammar, semantics, and even specialized knowledge such as medicine [2].

ChatGPT, an AI chatbot developed by OpenAI [3], was launched in November 2022 as an LLM [4,5]. Built on the generative pretrained transformer architecture, it uses tens of billions of parameters trained on massive internet text datasets [6]. ChatGPT excels at understanding and generating humanlike language, conducting natural dialogues, and delivering high-quality responses to user queries [7,8]. Its advanced text processing capabilities have driven unprecedented adoption: more than 1 billion monthly users within 4 months of release, demonstrating rapid societal integration [9].

ChatGPT demonstrates significant potential across diverse fields, such as translation, text summarization, and programming assistance [10]. Its effectiveness extends to specialized domains such as medical education [11]. In preclinical education, students use ChatGPT for medical knowledge acquisition and personalized learning [12,13]. Conversely, educators are able to use ChatGPT to implement innovative teaching methodologies and cultivate interactive learning environments [14-16]. In clinical education [17], ChatGPT simulates clinical environments to help students improve clinical skills [18-21]. Furthermore, the pass rates and accuracy in medical licensing exams and professional subject tests [22,23] in countries such as the United States [24-26], China [27,28], Japan [29,30], and Italy [31] have attracted significant attention [32]. ChatGPT is regarded as a significant instrument for promoting innovation and enhancing efficiency in the domain of medical education.

### Objectives

While existing literature reviews have explored ChatGPT’s applications and limitations in medical education, important questions remain unanswered. These include the collaboration networks among countries, institutions, and authors; the most influential journals; and the most cited publications. This study used bibliometric analysis to map collaboration networks and thematic evolution and provide a comprehensive understanding of the development of ChatGPT in medical education.

A bibliometric analysis is a rigorous scientific method that provides researchers across various fields with comprehensive guidance and support [33]. It allows researchers to gain in-depth insights into prevailing issues, key trends, and research limitations within their disciplines [34-36]. On the basis of recommendations from previous studies, this study proposes the following research questions (RQs):

1. Who are the most productive researchers and which are the most productive institutions and countries or regions in the field of ChatGPT in medical education?
2. What is the status of academic collaboration among researchers, countries, or regions in the field of ChatGPT in medical education?
3. Which are the most influential journals and articles in the field of ChatGPT in medical education?
4. What are the main research themes in the field of ChatGPT in medical education?
5. What are the research trends for ChatGPT in medical education?

## Methods

### Literature Sources and Search Strategy

The Web of Science database was chosen for this research due to its extensive coverage of more than 12,000 academic journals. When compared to other databases, including PubMed, MEDLINE, and Scopus, Web of Science offers a robust and reliable framework for bibliometric analysis [37]. After determining pertinent title keywords, a comprehensive bibliographic search was conducted online via the Web of Science database. The search was carried out in accordance with the following format:

((TS=(ChatGPT)) OR TS=(Chatbot*)) OR TS=(Chat Generative Pre-trained Transformer) and (((((((((((TS=(medic* educat*)) OR TS=(medic* student*)) OR TS=(clinical clerkship*)) OR TS=(medic* school*)) OR TS=(medic* learner*)) OR TS=(medic* trainee*)) OR TS=(medic*clerk*)) OR TS=(medical education)) OR TS=(medical student)) OR TS=(medical school)) OR TS=(medical student education)) OR TS=(healthcare). NOT ALL=(retracted)—Time: Tue Jul 01, 2025, 19:18:42 GMT+0800 (CST)

A total of 1817 documents were retrieved. These documents were screened according to the inclusion and exclusion criteria. The inclusion criteria were as follows: (1) original research articles and review articles related to ChatGPT in medical education and (2) English-language articles. After screening, of the 1817 retrieved articles, 1610 (88.61%) were retained. Following application of the exclusion criteria (articles unrelated to ChatGPT in medical education and duplicate articles), of the remaining 1610 articles, 1203 (74.72%) were excluded. The research topics of the 1203 excluded articles are summarized as follows: 370 (30.76%) were non-ChatGPT studies, 298 (24.77%) involved ChatGPT and patients, 263 (21.86%) involved ChatGPT and clinical treatment, 144 (11.97%) involved ChatGPT and hospitals, 52 (4.32%) involved ChatGPT and nonmedicine, 36 (2.99%) were ChatGPT non–medical education review articles, 20 (1.66%) involved ChatGPT and health care professional perspectives, 14 (1.16%) involved ChatGPT and nonmedical interactions with students, and 5 (0.42%) involved ChatGPT and veterinary medicine. This resulted in 407 publications being selected for bibliometric analysis. A comprehensive dataset, along with the corresponding references, was subsequently extracted from the relevant publications and organized in plain-text format for future research endeavors. This process was conducted independently by 2 authors, who cross-verified their work. Any discrepancies were resolved by a senior author.

### Data Collection and Statistics

The data were exported in plain-text file format using CiteSpace (version 6.3.R1; 64 bits; advanced) and R (version 4.5.0; R Foundation for Statistical Computing) with the *bibliometrix* package [38]. The data included the full record and cited references and were stored in the download format (.txt). The data extracted from the Bibliometric online platform [39] were exported in tab-delimited file format, with content and storage format identical to those described above.

CiteSpace, a bibliometric analysis software developed by Chaomei Chen, has achieved widespread use [40,41]. The software has been proven to provide feasible and reliable text mining and knowledge visualization methods. These methods have been used to explore research performance allocation and collaboration, research status and frontiers, and future trends. In this study, CiteSpace was used to detect parameters and visually analyze institution distribution, the dual-map overlay of journals, and burst detection.

Burst detection, based on the Kleinberg algorithm, uses an infinite state automaton to model document streams, thereby extracting meaningful structures [42]. These analyses can reveal themes exhibiting rapid growth over extended periods, as well as those that are inherently more transient.

VOSviewer, released in 2010 by Nees Jan van Eck and Ludo Waltman (Leiden University), is mainly used for bibliometric network graph analysis [43]. We used VOSviewer version 1.6.20 to visualize and analyze the country distribution and collaboration, journal distribution, author distribution and collaboration, and keyword distribution.

In addition, Bibliometrix (RTool of RStudio; Posit PBC) was used to visualize the distribution of keywords over time in the form of a heat map [44]. Microsoft Excel 2024 was used to analyze the monthly publication trends of literature from March 2023 to June 2025.

### Ethical Considerations

The study did not involve human participants, therefore the ethical approval was not required.

## Results

### Overview of Publication Status

**Figure 1.**
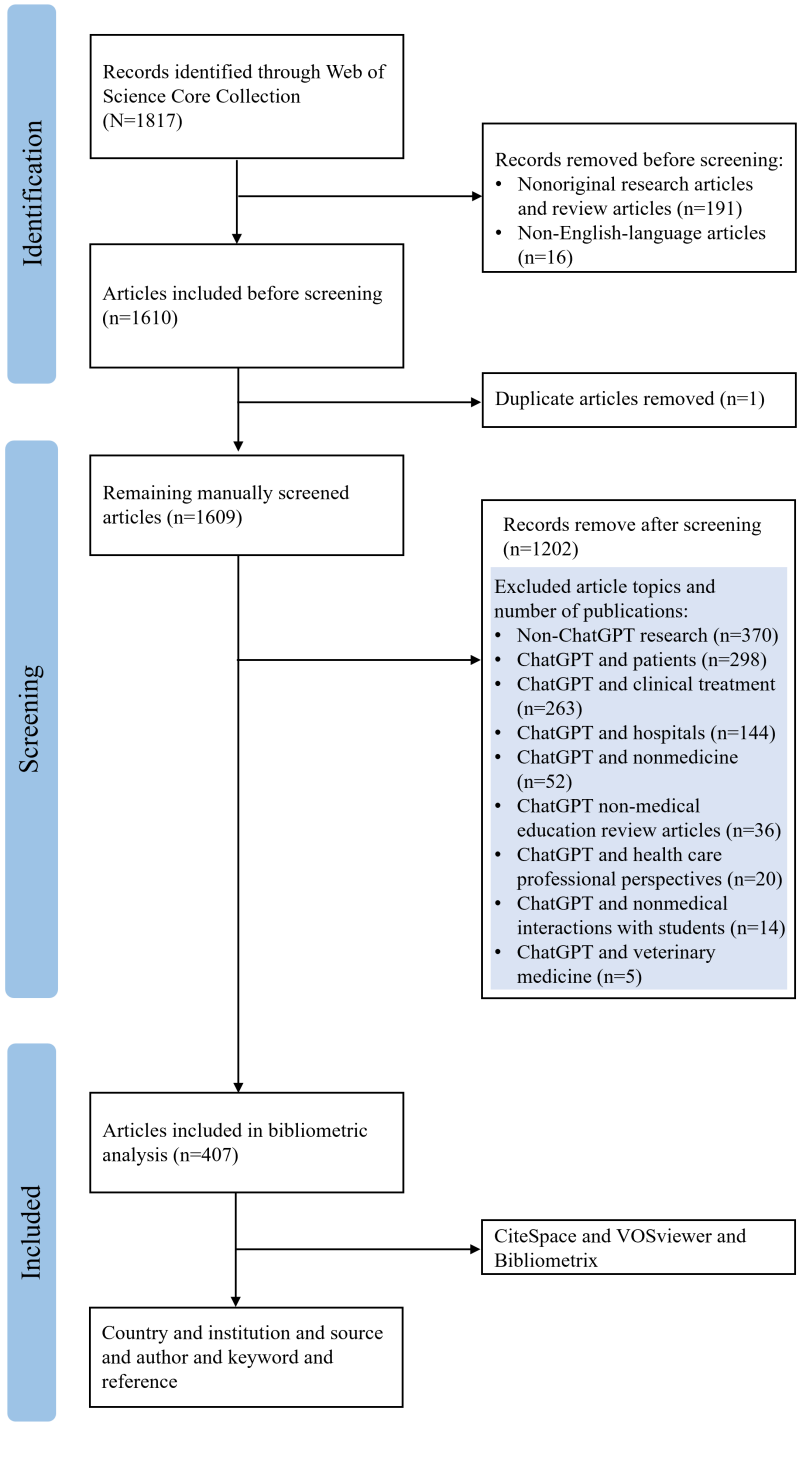
Flowchart of data collection and bibliometric analysis.

Our search and screening efforts yielded 407 articles (Figure 1). The release of the AI chatbot ChatGPT in November 2022 was immediately followed by 2 review articles on ChatGPT published in March 2023, which provided an overview of ChatGPT in medical education and prediction of potential future applications for ChatGPT. The analysis of publication trends for this topic was conducted using Microsoft Excel 2024 and presented the number of publications in tabular form (Table 1). The number of articles showed a gradual increasing trend. In early 2023, the number of published articles per month was less than 10, and starting in September 2023, this number increased significantly. By 2024, the number of articles per month remained at more than 10, and in 2025, it was more than 20 articles per month. In May 2025, the number of articles per month reached 33. This implies that, over time, an increasing number of scholars are focusing their attention on this domain.

**Table 1.** Number of articles per month and cumulative number.

Month and year	Monthly publications, n	Cumulative publications, n
March 2023	2	2
April 2023	1	3
May 2023	2	5
June 2023	2	7
July 2023	2	9
August 2023	8	17
September 2023	14	31
October 2023	13	44
November 2023	7	51
December 2023	12	63
January 2024	14	77
February 2024	15	92
March 2024	9	101
April 2024	15	116
May 2024	19	135
June 2024	17	152
July 2024	15	167
August 2024	15	182
September 2024	17	199
October 2024	20	219
November 2024	16	235
December 2024	24	259
January 2025	23	282
February 2025	23	305
March 2025	26	331
April 2025	22	353
May 2025	33	386
June 2025	21	407

### Analysis of National Publication Counts

**Figure 2.**
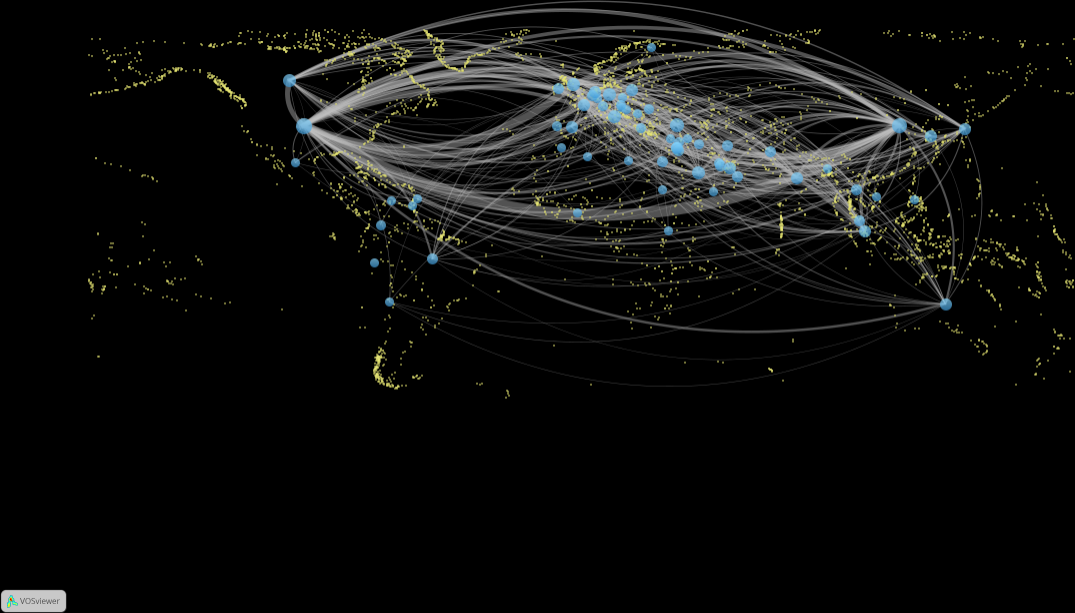
Countries and regions involved in the research in this field. The links between the countries and regions indicate their collaborations and connections.

Publication counts by country were used to analyze contributions in this field. According to the results, the publications originated from 66 countries. Visualizing the geographic distribution of the 66 countries using VOSviewer revealed that Asia, Europe, Africa, North America, South America, and Oceania were all represented and that the countries were mainly concentrated in the northern hemisphere (Figure 2). In total, 38% (25/66) of the countries were in Asia, and 33% (22/66) were from Europe, the 2 continents with the highest number of countries in this study. It is noteworthy that the linkages between countries or regions were concentrated between East Asia and North America, East Asia and Europe, North America and Europe, and North America and Oceania.

Table 2 shows the top 10 countries or regions in terms of number of publications and their corresponding citation frequency and centrality. The United States was the most prominent country with 31% (126/407) of the publications, closely followed by China, Türkiye, the United Kingdom, Canada, and Germany.

**Table 2.** Top 10 countries by number of publications and their number of citations and centrality (N=407).

Rank	Country	Publications, n (%)	Citations, n	Centrality
1	United States	126 (31)	3227	0.42
2	China	81 (19.9)	1026	0.19
3	Türkiye	35 (8.6)	215	0.08
4	United Kingdom	31 (7.6)	1844	0.17
5	Canada	24 (5.9)	668	0.05
6	Germany	22 (5.4)	463	0.06
7	Italy	19 (4.7)	730	0.06
8	Saudi Arabia	18 (4.4)	227	0.11
9	India	15 (3.7)	121	0.07
10	Japan	15 (3.7)	155	0.01

**Figure 3.**
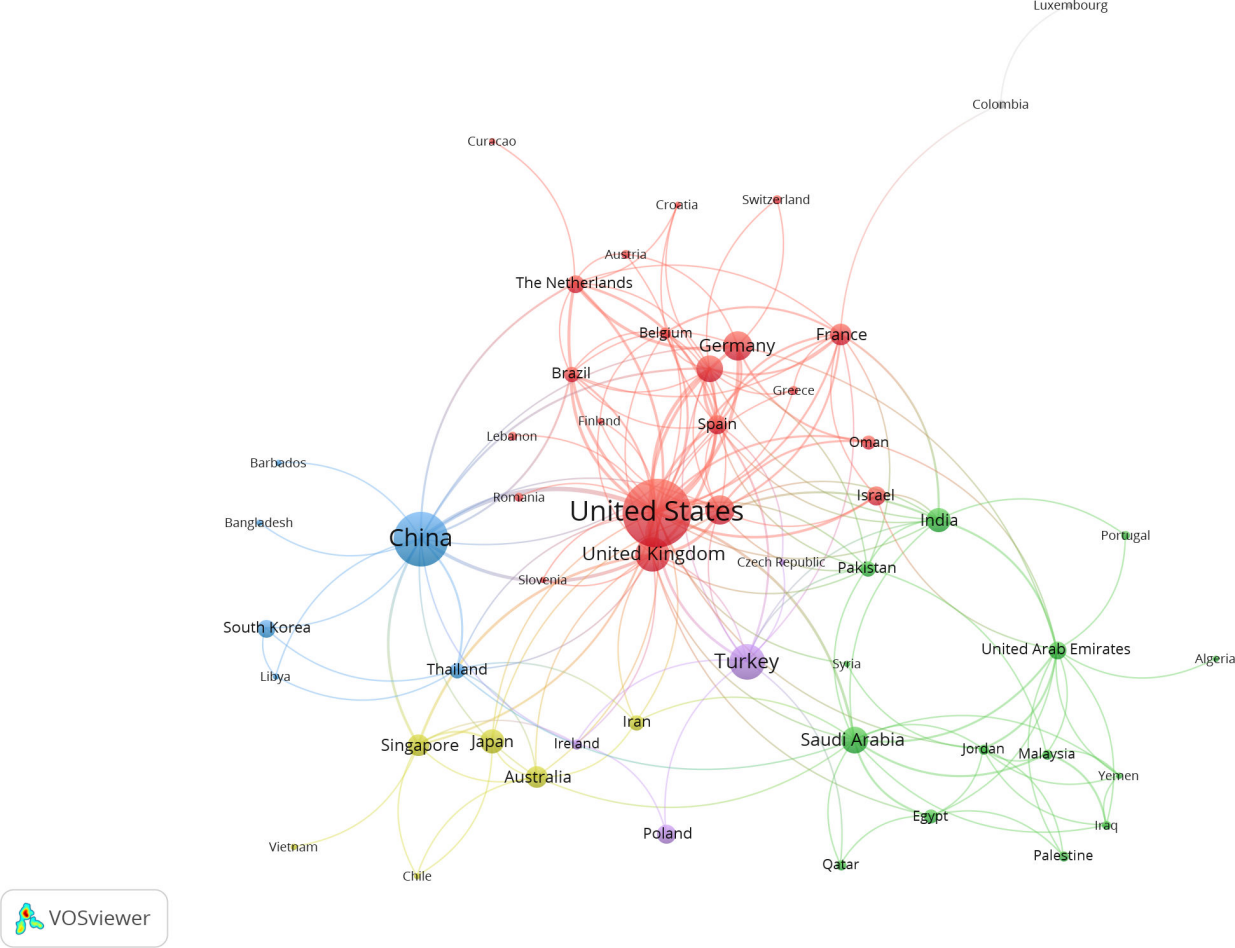
Analysis of collaborative network visualization of countries and regions in VOSviewer.

The results of the global collaboration network analysis show that countries and regions were roughly divided into 5 clusters in VOSviewer based on the closeness of collaboration and are indicated by different colors in Figure 3. The United States, China, Türkiye, the United Kingdom, and Canada were the top 5 countries in terms of the number of publications, and there were cooperative relationships between them. The betweenness centrality (BC) was calculated when analyzing the national and regional collaboration networks using CiteSpace, which represents the strength of association between nodes. Among the top 10 countries, the United States, China, the United Kingdom, and Saudi Arabia were the main research centers in this field.

### Analysis of Publication Institutions

**Figure 4.**
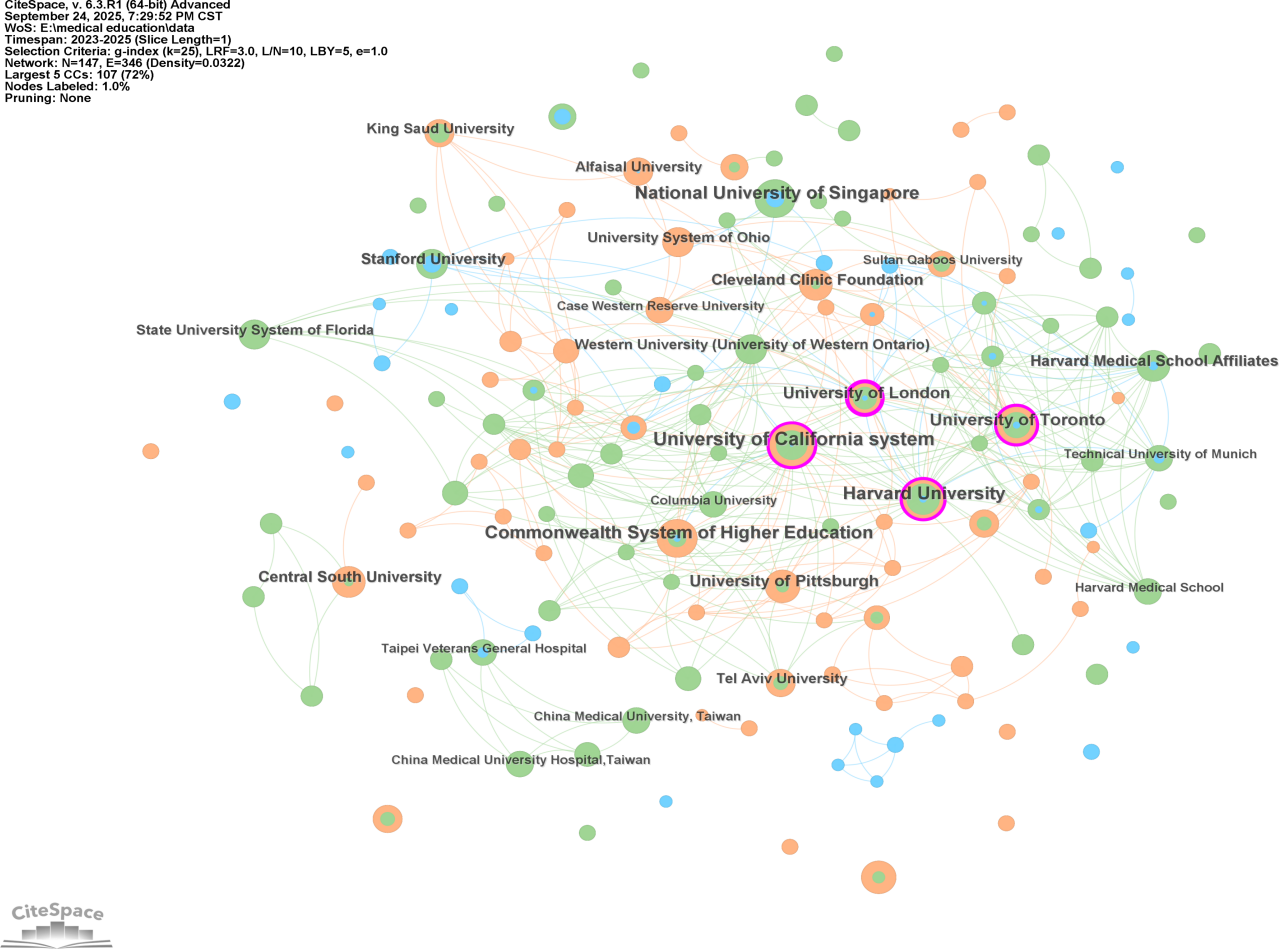
Analysis of collaborative network visualization of institutions in CiteSpace.

The scientific output came from 847 institutions. CiteSpace identified 147 institutions with 346 cooperative networks (Figure 4). The most productive institutions regarding research in this field were the University of California system (14/407, 3.4% of publications), Harvard University (11/407, 2.7% of publications), National University of Singapore (11/407, 2.7% of publications), the Commonwealth System of Higher Education (11/407, 2.7% of publications), the University of Toronto (10/407, 2.5% of publications), the University of London (8/407, 2% of publications), Gazi University (8/407, 2% of publications), the University of Pittsburgh (8/407, 2% of publications), Central South University (7/407, 1.7% of publications), and Stanford University (7/407, 1.7% of publications). Five of the top 10 institutions were from the United States. The remaining institutions were from Singapore, Canada, the United Kingdom, Türkiye, and China (Table 3).

**Table 3.** Top 10 institutions and their centrality in CiteSpace (N=407).

Rank	Institution	Publications, n (%)	Centrality
1	University of California system	14 (3.4)	0.17
2	Harvard University	11 (2.7)	0.14
3	National University of Singapore	11 (2.7)	0
4	Commonwealth System of Higher Education	11 (2.7)	0.03
5	University of Toronto	10 (2.5)	0.1
6	University of London	8 (2)	0.22
7	Gazi University	8 (2)	0
8	University of Pittsburgh	8 (2)	0.01
9	Central South University	7 (1.7)	0
10	Stanford University	7 (1.7)	0.08

In CiteSpace, each node represents a institution, and the radius of a node increases with its contribution to research in the field, whereas the BC is proportional to the size of the purple ring around the nodes. The larger the purple circle, the larger the value of the betweenness centrality. Network visualization revealed that there were 4 central institutions: the University of California system (BC=0.17), Harvard University (BC=0.14), the University of Toronto (BC=0.10), and the University of London (BC=0.22; Figure 4). This reflects the significant bridging role of these institutions in the research on ChatGPT in medical education.

### Analysis of Publication Quantity and Journal Impact

**Figure 5.**
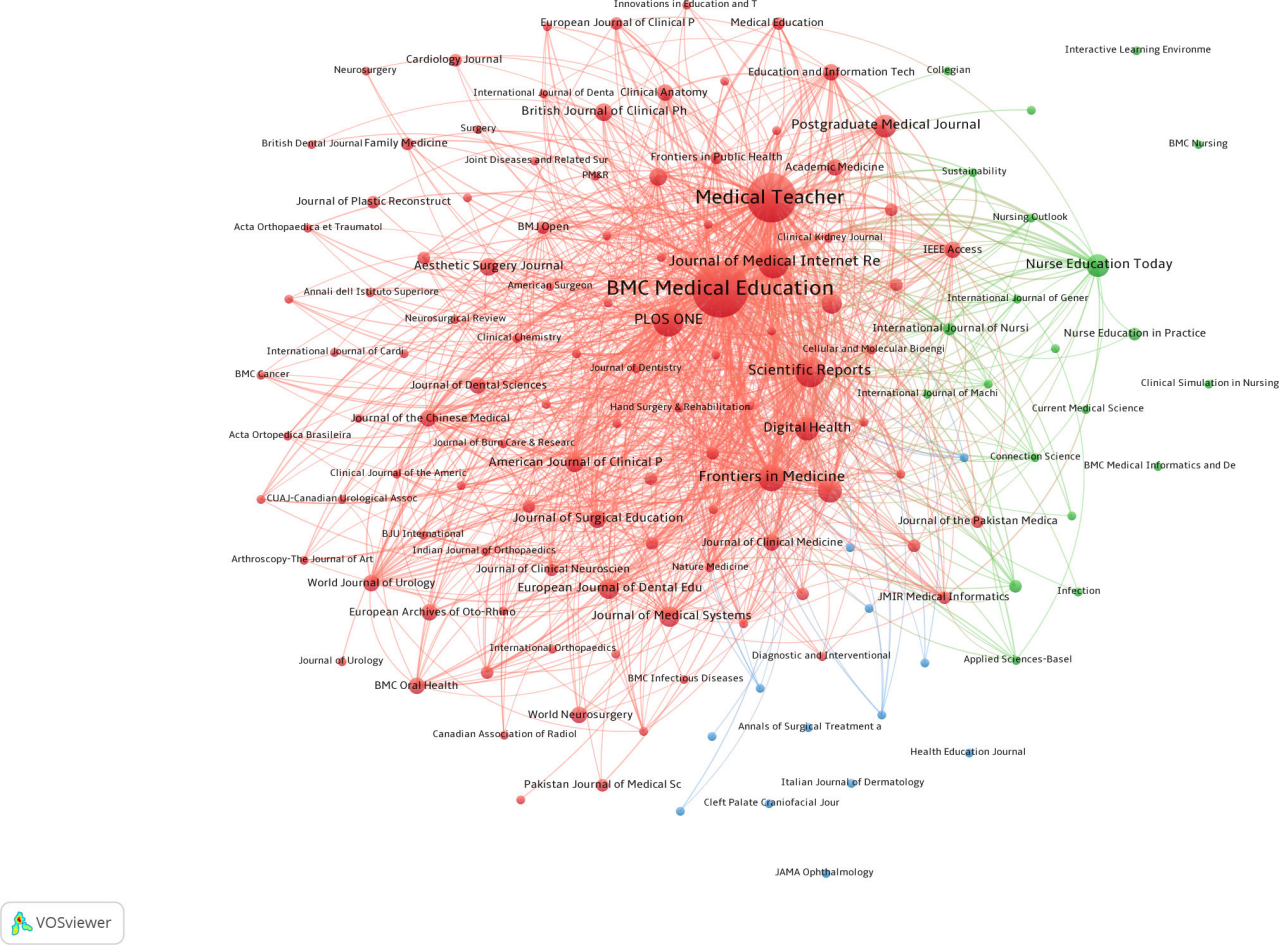
Analysis of collaborative network visualization of journals in VOSviewer.

**Figure 6.**
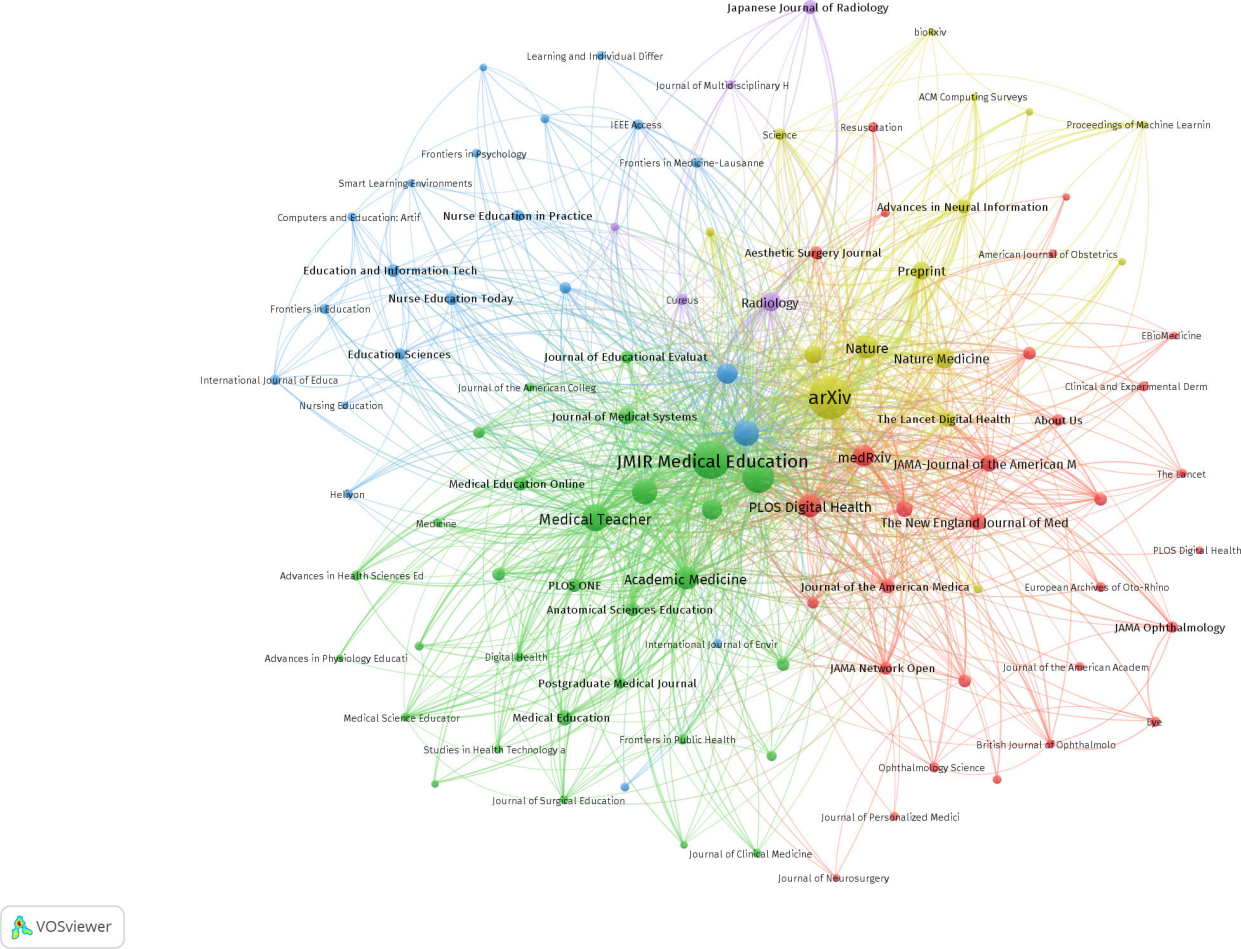
Analysis of collaborative network visualization of journals’ citations in VOSviewer.

This study encompassed 407 articles published across 197 sources and journals. Table 4 lists the 10 most prolific sources and journals ranked by publication volume, along with their 2024 impact factor (IF). The top 10 journals published a total of 139 papers. Among these, *BMC Medical Education* (IF of 3.2; quartile 1; 40/407, 9.8% of publications) had the highest number of publications, followed by *Medical Teacher* (IF of 4.4; quartile 1; 32/407, 7.9% of publications), the *Journal of Medical Internet Research* (IF of 6.0; quartile 1; 11/407, 2.7% of publications), *Scientific Reports* (IF of 3.9; quartile 1; 11/407, 2.7% of publications), and *PLOS ONE* (IF of 2.6; quartile 2; 10/407, 2.5% of publications). Eight of the top 10 journals in terms of publications were distributed in quartile 1 of the Journal Citation Reports (Figure 5). The source or journal with the highest cocitation frequency was *arXiv*, followed by *JMIR Medical Education*, *Cureus*, *Medical Teacher*, *BMC Medical Education*, and the *Journal of Medical Internet Research* (Figure 6). Seven of the top 10 sources or journals in terms of cocitation frequency were distributed in quartile 1 of the Journal Citation Reports (Table 5). It is important to note that 3 of the top 10 journals in terms of publications were also among the top 10 journals in terms of cocitation frequency: *Medical Teacher*, *BMC Medical Education*, and the *Journal of Medical Internet Research*.

**Table 4.** Top 10 sources by number of publications and their corresponding journal impact factor (IF; Journal Citation Reports [JCR] 2024) and JCR quartile (N=407).

Rank	Source	Publications, n (%)	IF (JCR 2024)	JCR quartile
1	*BMC Medical Education*	40 (9.8)	3.2	1
2	*Medical Teacher*	32 (7.9)	4.4	1
3	*Journal of Medical Internet Research*	11 (2.7)	6.0	1
4	*Scientific Reports*	11 (2.7)	3.9	1
5	*PLOS ONE*	10 (2.5)	2.6	2
6	*Frontiers in Medicine*	9 (2.2)	3.0	1
7	*Digital Health*	7 (1.7)	3.3	1
8	*Healthcare*	7 (1.7)	2.7	2
9	*Nurse Education Today*	6 (1.5)	4.2	1
10	*Postgraduate Medical Journal*	6 (1.5)	2.7	1

**Table 5.** Top 10 sources by number of cocitations and their corresponding journal impact factor (IF; Journal Citations Report [JCR] 2024) and JCR quartile.

Rank	Source	Number of cocitations	IF (JCR 2024)	JCR quartile
1	*arXiv*	692	None	None
2	*JMIR Medical Education*	542	3.2	1
3	*Cureus Journal of Medical Science*	357	1.3	2
4	*Medical Teacher*	252	4.4	1
5	*BMC Medical Education*	237	3.2	1
6	*Journal of Medical Internet Research*	216	6.0	1
7	*PLOS Digital Health*	191	7.7	1
8	*Nature*	188	48.5	1
9	*Academic Medicine*	187	5.2	1
10	*medRxiv*	164	None	None

The visualization in VOSviewer showed the journals that have published articles on ChatGPT in medical education and the relationship between them. On the basis of the similarity between journals, they were divided into 3 categories: the red cluster focused on educational, technical, and basic research (eg, *BMC Medical Education* and *Medical Teacher*); the green cluster focused on the discipline of nursing and extended to nursing education, clinical simulation training, and health care informatics (eg, *Nurse Education Today* and *International Journal of Nursing Studies*); and the blue cluster focused on specialized clinical practice, particularly in surgery, obstetrics and gynecology, orthopedics, and ophthalmology (eg, *American Journal of Obstetrics and Gynecology* and *Cleft Palate Craniofacial Journal*).

**Figure 7.**
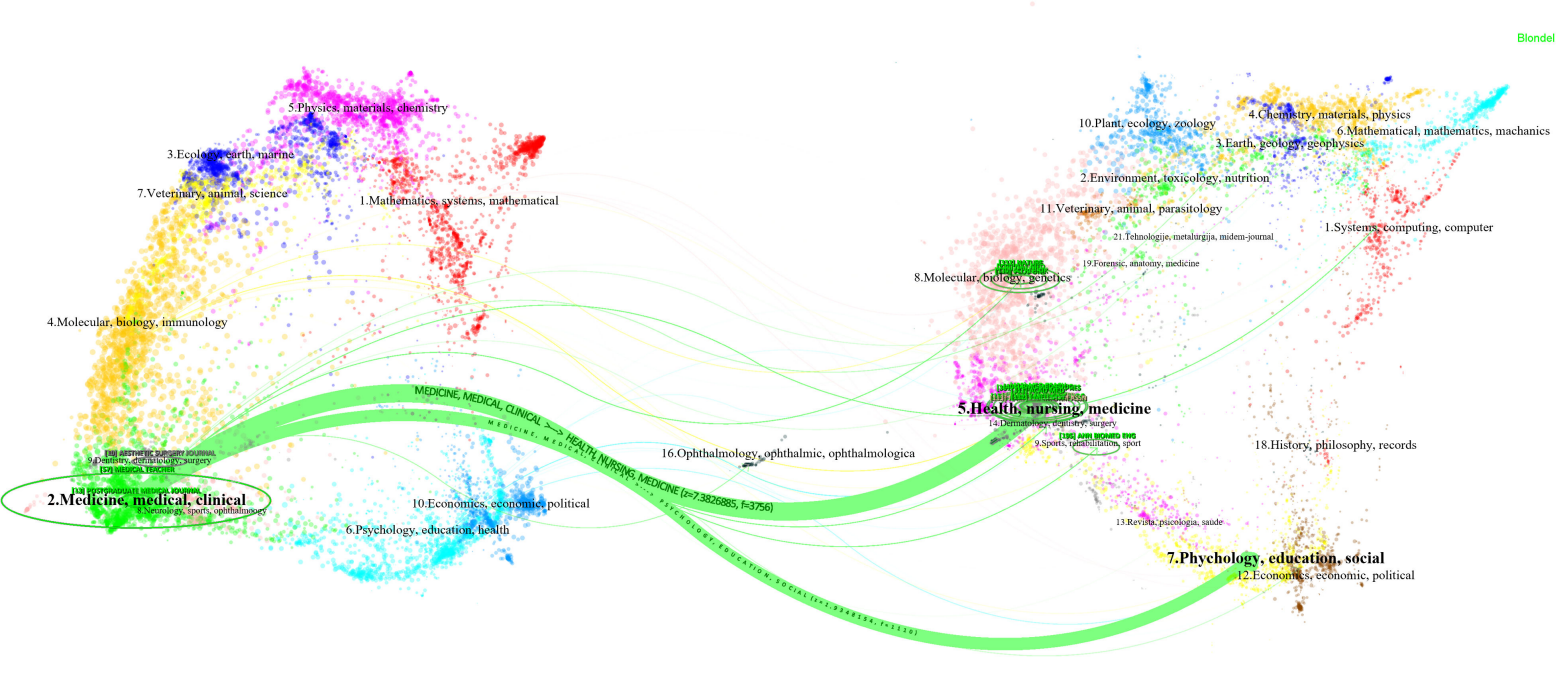
The dual-map overlay of journals.

Journals were among the most common sources for publishing research results. Figure 7 presents a dual-map overlay of all academic journals, illustrating the citation paths of various subject areas. This dual-map overlay consists of 2 base maps: one on the left for citing journals and one on the right for cited journals [45]. The disciplines represented by the citing journals are indicated by the labels on the left side of the dual map, whereas the disciplines of the cited journals are shown on the right [46].

Notably, 2 primary citation trajectories can be identified on the map. They are from health, nursing, and medicine to medicine, medical, and clinical (green) and from psychology, education, and social to medicine, medical, and clinical (green). In addition, the leading-edge research results were mainly distributed in 2 (medicine, medical, and clinical). This suggests that research on ChatGPT in medical education is particularly active within these disciplines. In contrast, the knowledge base that frontier researchers rely on primarily stems from 5 (health, nursing, and medicine) and 7 (psychology, education, and social). Citing journals and cited journals were both from the field of medicine. This implies that the application of ChatGPT in fields where medical education intersects with other disciplines is still limited.

### Analysis of the Author Collaboration Network Graph

**Figure 8.**
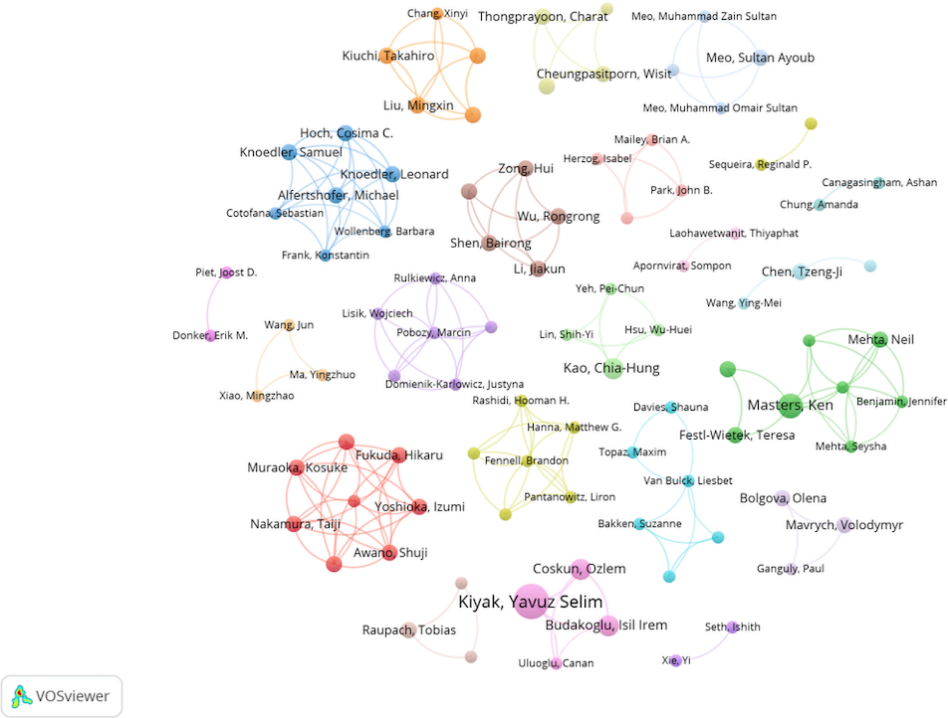
Collaborative network visualization of authors in VOSviewer.

Analyzing the coauthorship network of this study helped identify potential collaborators and authoritative figures in the field. The author with the highest number of publications was Yavuz Selim Kiyak (Table 6). He has formed a stable core collaboration group with Isil Irem Budakoglu and Ozlem Coskun, who are from the same institution (Figure 8). All 3 authors ranked among the top 10 in terms of publication volume. However, among the top 10 most prolific authors, the remaining 7 have each formed independent teams. The analysis of author collaboration suggests that most academic research was conducted in independent teams without cross-team communication. Therefore, large interinstitutional collaboration networks have yet to be established.

**Table 6.** Top 10 authors by number of publications and their institutions and total link strength (N=407).

Rank	Author	Publications, n (%)	Institution	Total link strength
1	Yavuz Selim Kiyak	8 (2)	Gazi University (Türkiye)	1372
2	Ken Masters	5 (1.2)	Sultan Qaboos University (Oman)	945
3	Isil Irem Budakoglu	4 (1)	Gazi University (Türkiye)	1081
4	Ozlem Coskun	4 (1)	Gazi University (Türkiye)	837
5	Chia-Hung Kao	4 (1)	China Medical University (China)	641
6	Michael Alfertshofer	3 (0.7)	Ludwig Maximilian University of Munich (Germany)	774
7	Shuji Awano	3 (0.7)	Kyushu Dental University (Japan)	748
8	Olena Bolgova	3 (0.7)	Alfaisal University (Saudi Arabia)	735
9	Tzeng-Ji Chen	3 (0.7)	Taipei Veterans General Hospital, Hsinchu Branch (China)	1041
10	Wisit Cheungpasitporn	3 (0.7)	Mayo Clinic (United States)	1087

Co-citation refers to the situation in which different authors are cited by the same article. These authors then form a co-citation relationship.The increase in co-citation counts indicates a greater degree of similarity among different authors' research, with the analysis itself reflecting the research strength of the respective authors. Table 7 lists the top 10 authors in terms of cocitation frequency. The most frequently cocited authors were Tiffany H Kung (n=176), Aidan Gilson (n=141), Malik Sallam (n=109), and Arun James Thirunavukarasu (n=58). It is noteworthy that Kung is highly influential in the field of research on ChatGPT in medical education.

**Table 7.** Top 10 authors by number of citations and their institutions and total link strength.

Rank	Author	Number of citations	Institution	Total link strength
1	Tiffany H Kung	176	Harvard Medical School (United States)	1945
2	Aidan Gilson	141	Yale School of Medicine (United States)	1577
3	Malik Sallam	109	The University of Jordan (Jordan)	1264
4	Arun James Thirunavukarasu	58	University of Cambridge (United Kingdom)	773
5	Gunther Eysenbach	53	JMIR Publications (Canada)	645
6	Hyunsu Lee	49	Keimyung University (South Korea)	775
7	Karan Singhal	49	Google Research (United States)	826
8	Yavuz Selim Kıyak	42	Gazi University (Türkiye)	389
9	Andrew Mihalache	42	University of Western Ontario (Canada)	549
10	Rehan Ahmed Khan	40	Riphah International University (Pakistan)	502

### Keyword Analysis of Global Research

**Figure 9.**
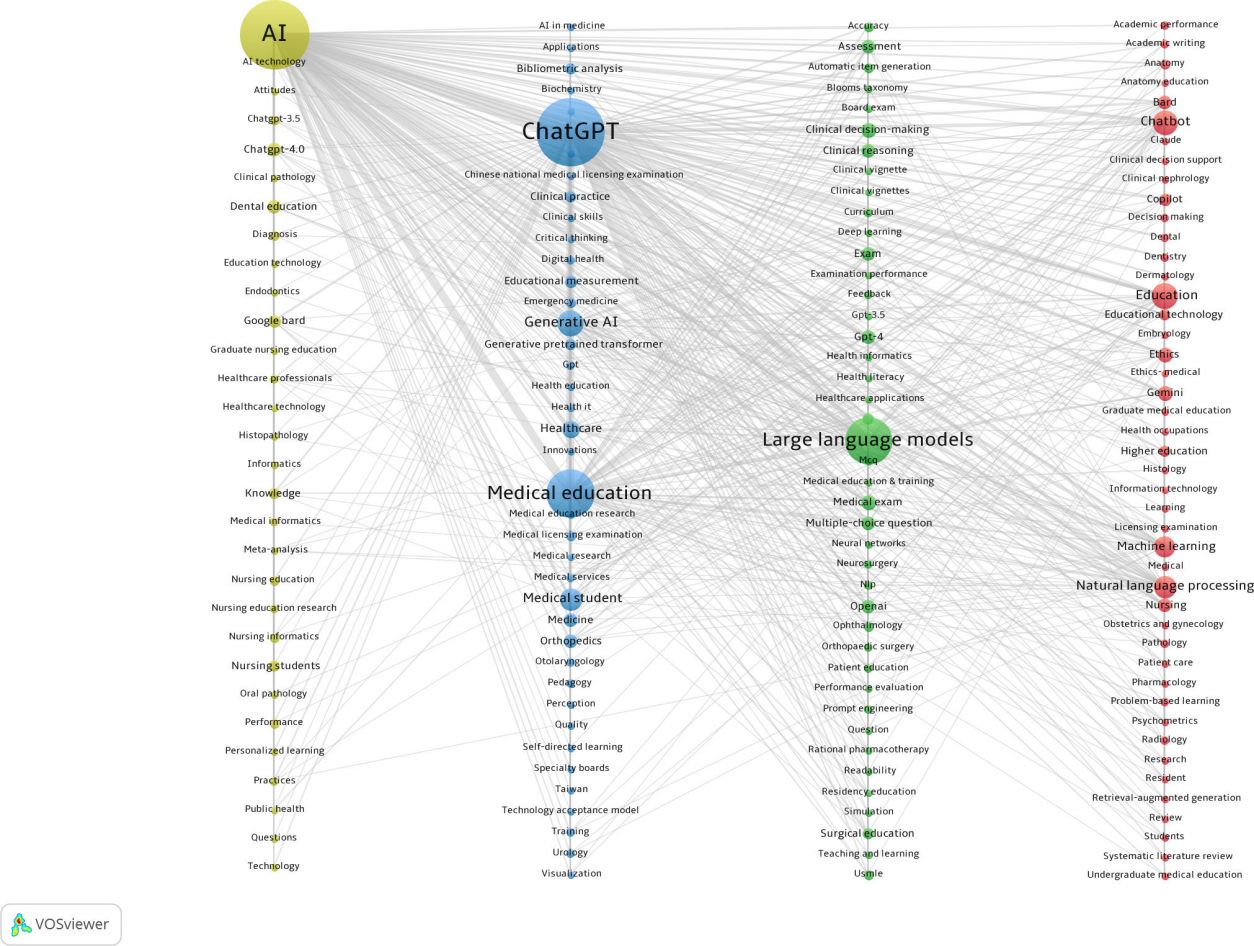
The co-occurrence of keywords in VOSviewer.

To provide an overview of the primary content of the articles, it is possible to use keywords to analyze the frontiers of research on ChatGPT in medical education. Table 8 lists the top 20 keywords by frequency. The most frequent keyword was “artificial intelligence (AI),” followed by “ChatGPT,” “medical education,” “large language models,” and “generative AI.” The keyword co-occurrence network was visualized using VOSviewer, where the connecting lines between different keywords indicate that they have co-occurrence relationships (Figure 9). The keywords that make up this network were categorized into 4 clusters. The keywords in the red cluster in Figure 9 were related to the foundational elements of medical education, core disciplines, and ethical issues, such as “academic writing,” “radiology,” “ethics-medical,” and “machine learning.” The keywords in the green cluster focused on assessment methods, exam systems, and clinical decision-making processes in medical education, such as “medical exam,” “clinical decision-making,” and “teaching and learning.” The keywords in the blue cluster focused on specific applications, challenges, and practical effects of generative AI in medical practice, medical education, and specialties, such as “clinical practice,” “nursing education,” and “clinical skills.” The keywords in the yellow cluster were related to the evaluation of different generative AI models and multiple research methods and practices, such as “google bard,” “meta-analysis,” and “diagnosis.”

**Table 8.** Top 20 keywords with the highest frequency of occurrence and their corresponding total link strength.

Rank	Keyword	Number of occurrences	Total link strength
1	“AI”	225	715
2	“ChatGPT”	216	676
3	“Medical education”	107	336
4	“Large language models”	101	338
5	“Generative AI”	29	108
6	“Education”	28	102
7	“Chatbot”	26	86
8	“Natural language processing”	22	110
9	“Medical student”	20	56
10	“Machine learning”	18	74
11	“Healthcare”	12	62
12	“Ethics”	9	48
13	“Gemini”	9	39
14	“Clinical decision-making”	9	26
15	“Medical exam”	9	48
16	“Bard”	8	40
17	“Nursing”	8	31
18	“Assessment”	8	40
19	“Clinical reasoning”	8	20
20	“Exam”	8	34

**Figure 10.**
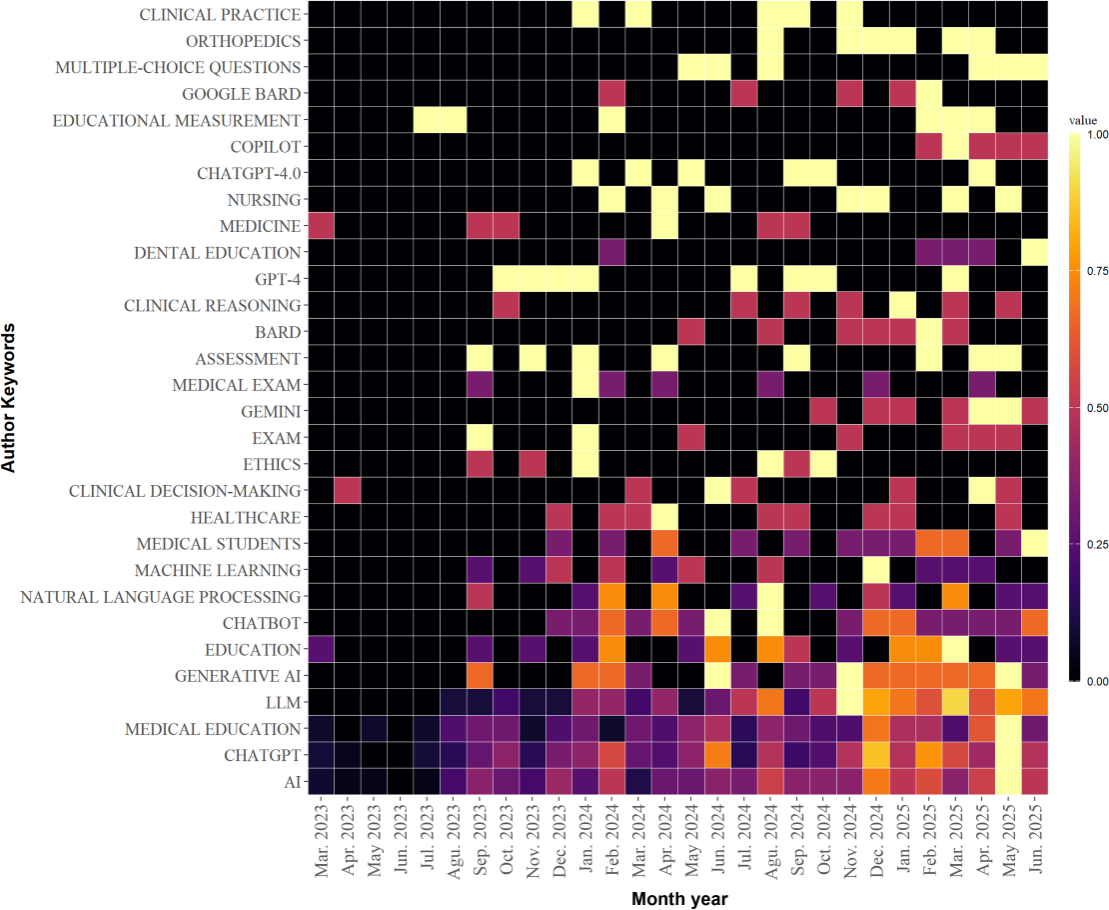
Monthly distribution heat map of keywords in Bibliometrix.

**Figure 11.**
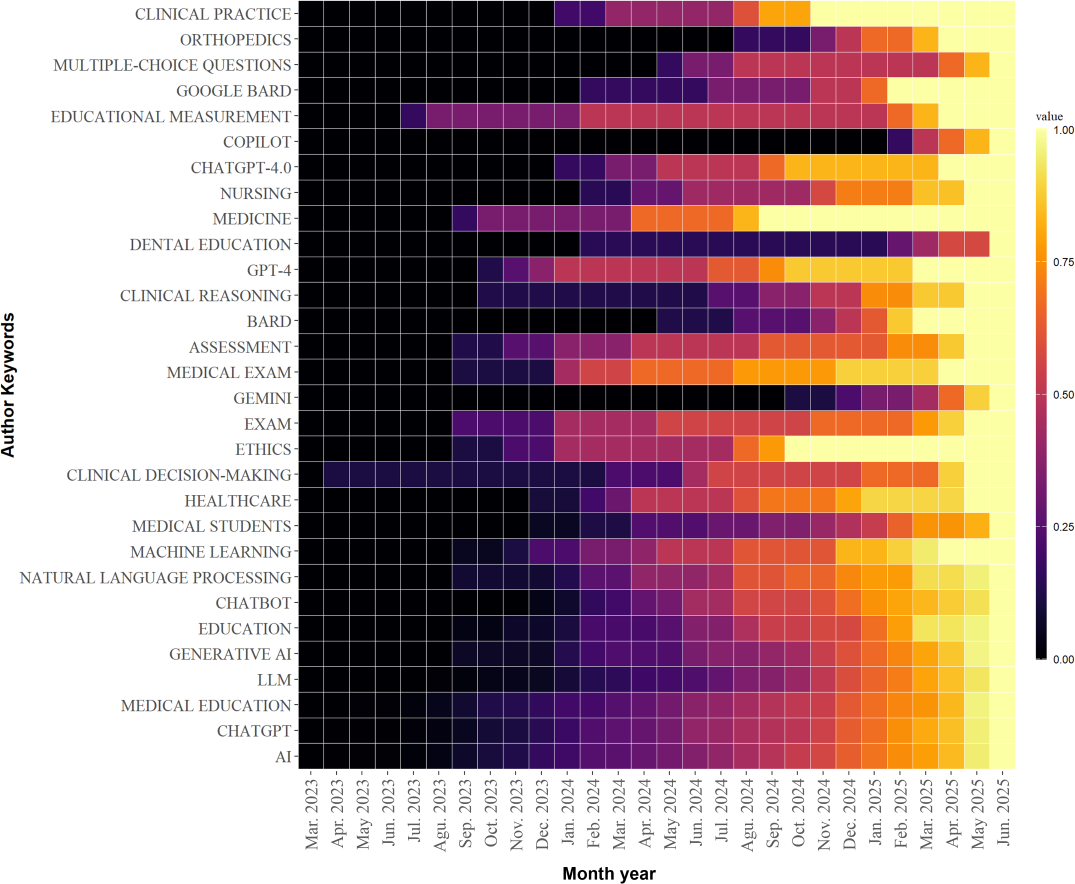
Cumulative distribution heat map of keywords in Bibliometrix.

Figure 10 shows the monthly prevalence of the keywords from March 2023 to June 2025. Keywords such as “educational evaluation” and “medical disciplines” were research hot spots in 2023. Studies on GPT-4 continued throughout 2024. By 2025, research expanded to other LLMs such as Google Bard, Gemini, and Copilot. Research on medical exams continued from 2024 to 2025. Figure 11 shows the cumulative frequency of keywords between March 2023 and June 2025. Although research on clinical decision-making began in April 2023, related studies increased nearly in 2025. Ethics, starting in early 2024, rapidly became a research hot spot in a relatively short period.

### Characteristics of Cited Research Articles

Table 9 lists the top 10 articles in terms of citation frequency. The most frequently cited article was “Performance of ChatGPT on USMLE: Potential for AI-Assisted Medical Education Using Large Language Models” [47] (n=169). The second most cited article was “How Does ChatGPT Perform on the United States Medical Licensing Examination (USMLE)? The Implications of Large Language Models for Medical Education and Knowledge Assessment” [48] (n=125). Both articles were about ChatGPT’s participation in the United States Medical Licensing Examination (USMLE). They evaluated ChatGPT’s performance on the USMLE, reflecting strong researcher interest in AI’s exam capabilities during the 2023 to 2025 study period.

**Table 9.** Top 10 most cited references.

Rank	Article title	Source	Authors	Year	Number of citations
1	“Performance of ChatGPT on USMLE: Potential for AI-Assisted Medical Education Using Large Language Models”	*PLOS Digital Health*	Kung et al [47]	2023	169
2	“How Does ChatGPT Perform on the United States Medical Licensing Examination (USMLE)? The Implications of Large Language Models for Medical Education and Knowledge Assessment”	*JMIR Medical Education*	Gilson et al [48]	2023	125
3	“ChatGPT Utility in Healthcare Education, Research, and Practice: Systematic Review on the Promising Perspectives and Valid Concerns”	*Healthcare (Basel)*	Malik Sallam [49]	2023	63
4	“The Rise of ChatGPT: Exploring Its Potential in Medical Education”	*Anatomical Sciences Education*	Hyunsu Lee [50]	2024	51
5	“The Role of ChatGPT, Generative Language Models, and Artificial Intelligence in Medical Education: A Conversation With ChatGPT and a Call for Papers”	*JMIR Medical Education*	Gunther Eysenbach [19]	2023	51
6	“Large Language Models in Medicine”	*Nature Medicine*	Thirunavukarasu et al [51]	2023	44
7	“ChatGPT - Reshaping Medical Education and Clinical Management”	*Pakistan Journal of Medical Sciences*	Khan et al [52]	2023	39
8	“Artificial Hallucinations in ChatGPT: Implications in Scientific Writing”	*Cureus Journal of Medical Science*	Alkaissi et al [53]	2023	36
9	“ChatGPT in Medicine: An Overview of Its Applications, Advantages, Limitations, Future Prospects, and Ethical Considerations”	*Frontiers in Artificial Intelligence*	Dave et al [54]	2023	35
10	“Benefits, Limits, and Risks of GPT-4 as an AI Chatbot for Medicine”	*New England Journal of Medicine*	Lee et al [55]	2023	34

Table 10 shows the top 20 references with the strongest citation bursts. The first citation burst occurred in 2023. This was a study comparing ChatGPT and Korean medical students on a parasitology exam. An article published in 2023, titled “Chat Generative Pretrained Transformer Fails the Multiple-Choice American College of Gastroenterology Self-Assessment Test” [56], experienced a citation burst in 2024, with the burst lasting until 2025. Researchers have continued to study ChatGPT’s ability to pass medical exams; the types of exams have ranged from the USMLE to basic subject exams. Furthermore, researchers have begun to evaluate ChatGPT’s performance on different types of exam questions.

**Table 10.** Top 20 references with the strongest citation bursts.

Title	First author	Source	IF2024	JCR	Publication type	Publication year	Strength	Begin	End	2023‐2025
Are ChatGPT’s Knowledge and Interpretation Ability Comparable to Those of Medical Students in Korea for Taking a Parasitology Examination?: A Descriptive Study	Sun Huh [57]	Journal of Educational Evaluation for Health Professions	3.7	Q1	Article	2023	3.97	2023	2023	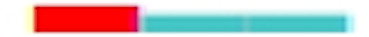
Will ChatGPT Transform Healthcare?	No authors listed	Nature Medicine	50	Q1	Editorial	2023	3.91	2023	2023	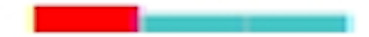
ChatGPT Passing USMLE Shines a Spotlight on the Flaws of Medical Education	Amarachi B Mbakwe [58]	PLOS Digital Health	7.7	Q1	Editorial	2023	3.79	2023	2023	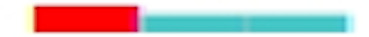
ChatGPT: the Future of Discharge Summaries?	Sajan B Patel [59]	The Lancet Digital Health	24.1	Q1	Comment	2023	3.54	2023	2023	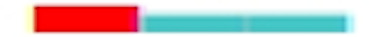
ChatGPT for Clinical Vignette Generation, Revision, and Evaluation	James RA Benoit [60]	medRxiv	None	None	Article	2023	3.35	2023	2023	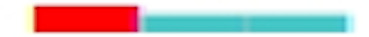
Abstracts Written by ChatGPT Fool Scientists	Holly Else [61]	Nature	48.5	Q1	Article	2023	3.09	2023	2023	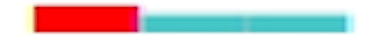
Benefits, Limits, and Risks of GPT-4 as an AI Chatbot for Medicine	Peter Lee [55]	The New England Journal of Medicine	78.5	Q1	Review	2023	3.01	2023	2023	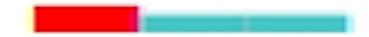
Tools Such as ChatGPT Threaten Transparent Science; Here Are Our Ground Rules for Their Use	No authors listed	Nature	48.5	Q1	Editorial	2023	2.23	2023	2023	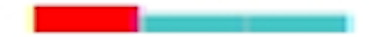
Could AI Help You to Write Your Next Paper?	Matthew Hutson [62]	Nature	48.5	Q1	Review	2022	2.23	2023	2023	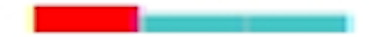
Appropriateness of Cardiovascular Disease Prevention Recommendations Obtained From a Popular Online Chat-Based Artificial Intelligence Model	Ashish Sarraju [63]	JAMA-Journal of the American Medical Association	55	Q1	Article	2023	2.23	2023	2023	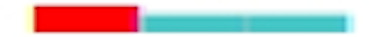
Medical Education Trends for Future Physicians in the Era of Advanced Technology and Artificial Intelligence: An Integrative Review	Eui-Ryoung Han [64]	BMC Medical Education	3.2	Q1	Review	2019	2.23	2023	2023	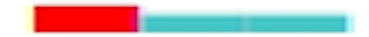
The Exciting Potential for ChatGPT in Obstetrics and Gynecology	Amos Grünebaum [65]	American Journal of Obstetrics and Gynecology	8.4	Q1	Article	2023	2.23	2023	2023	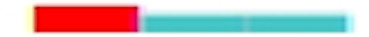
ChatGPT: Not All Languages Are Equal	Mohamed L Seghier [66]	Nature	48.5	Q1	Comment	2023	2.23	2023	2023	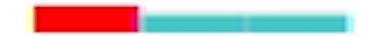
GPT Takes the Bar Exam	Michael James Bommarito [67]	arXiv	None	None	Article	2022	2.23	2023	2023	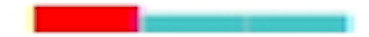
Performance of ChatGPT on USMLE: Potential for AI-Assisted Medical Education Using Large Language Models	Tiffany H Kung [47]	PLOS Digital Health	7.7	Q1	Article	2023	2.13	2023	2023	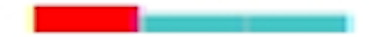
ChatGPT: Five Priorities for Research	Eva AM van Dis [8]	Nature	48.5	Q1	Comment	2023	2.13	2023	2023	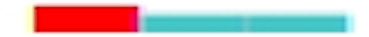
Chat Generative Pretrained Transformer Fails the Multiple-Choice American College of Gastroenterology Self-Assessment Test	Kelly Suchman [56]	American Journal of Gastroenterology	7.6	Q1	Article	2023	1.8	2024	2025	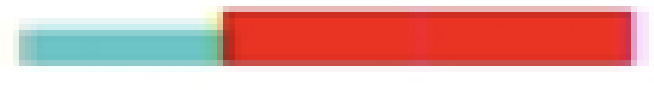
Capabilities of GPT-4 on Medical Challenge Problems	Harsha Nori [68]	arXiv	None	None	Article	2023	1.67	2023	2023	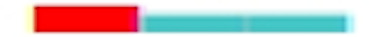
Domain-Specific Language Model Pretraining for Biomedical Natural Language Processing	Yu Gu [69]	ACM Transactions on Computing for Healthcare	8	Q1	Article	2022	1.67	2023	2023	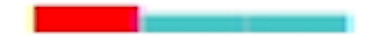
Natural Language Processing: State of the Art, Current Trends and Challenges	Diksha Khurana [70]	Multimedia Tools and Applications	3	Q3	Review	2023	1.67	2023	2023	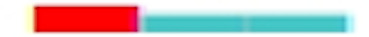

**Figure 12.**
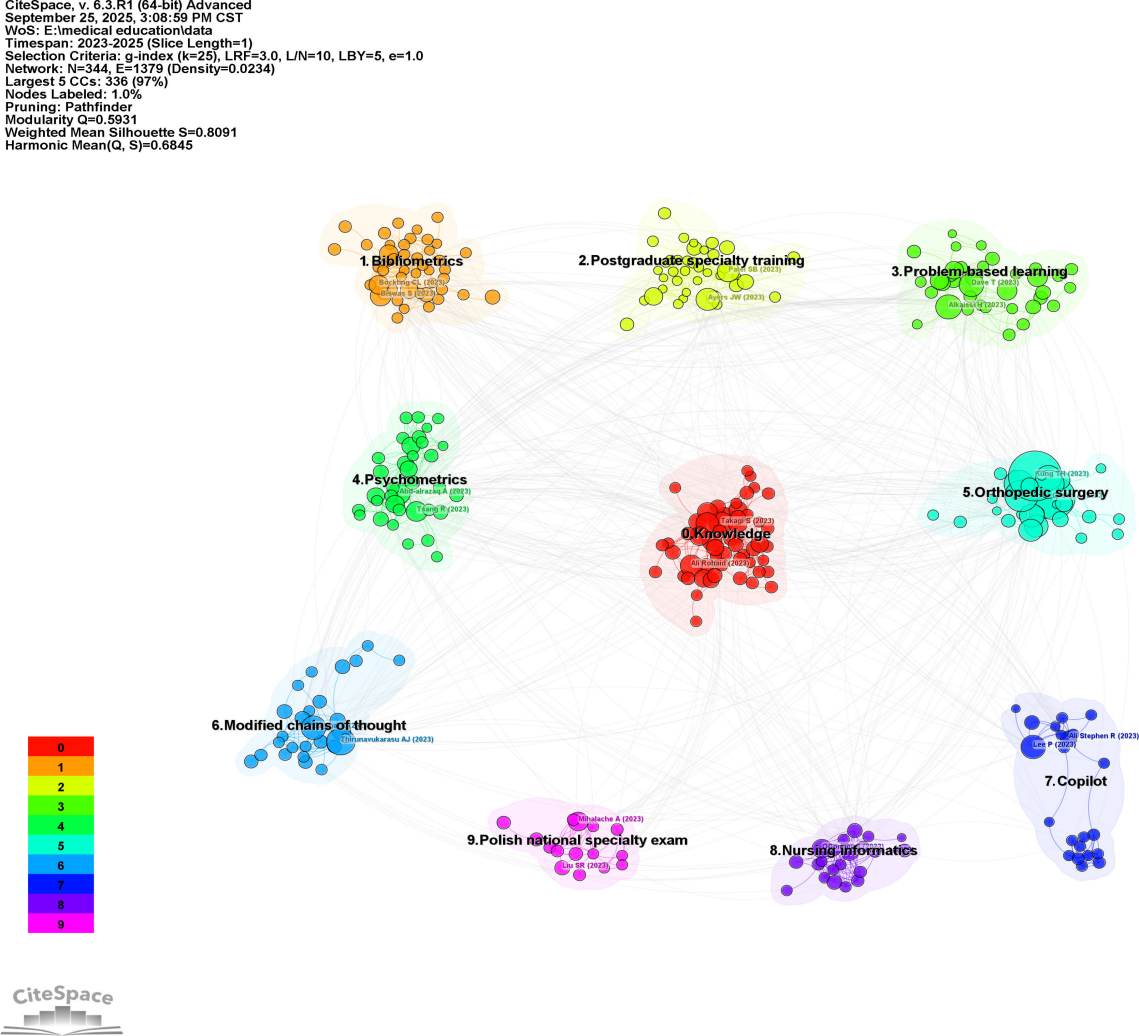
Clustering of references based on similarity.

The process of clustering analysis was conducted based on the relevance between documents, with the result that the literature was divided into 9 categories (Figure 12), each of which was identified with a different color. The category with the highest number of publications was cluster 0. The term “knowledge” was a frequently occurring keyword in this category, indicating a concentration of studies evaluating ChatGPT’s medical knowledge. This finding is related to the research themes of the highly cited articles and burst articles. The cluster evolution shows that cluster 0 (knowledge) originated from clusters 2 (postgraduate specialty training) and 3 (problem-based learning), later developing into cluster 5 (orthopedic surgery). This progression reflects a research shift from foundational knowledge toward clinical applications. It is noteworthy that, after Microsoft released Copilot in May 2023, studies expanded to cluster 7 (Copilot).

## Discussion

### Principal Findings

We analyzed Web of Science literature on ChatGPT in medical education using VOSviewer and CiteSpace. The bibliometric results showed that researchers from 66 countries have participated in this field. The United States was the most prolific contributor, with 31% (126/407) of the published papers included in this study. Furthermore, the University of California system was ranked first with 3.4% (14/407) of the publications, reflecting the sustained investment of the United States in this area. Networks of countries and institutions have been established. This confirms active global communication and cooperation on the application of ChatGPT in medical education. Current research collaborations facilitate deeper international exchange in this field.

Notable contributors in this field include Yavuz Selim Kiyak, Ken Masters, Isil Irem Budakoglu, and Ozlem Coskun, all of whom are from academic institutions. Notably, 2 of the top 10 authors with the most publications are from hospitals or clinics. This indicates that clinicians are increasingly paying attention to the application of ChatGPT in clinical education. The results of the journal analysis showed that both citing and cited journals were from the field of medicine, and there was no emerging trend toward interdisciplinary research. The results of keyword analysis showed a shift in research focus from broader topics such as medical education assessment and medical exams to specific clinical disciplines such as dentistry and nephrology.

### Comparison to the Literature

Our research findings are consistent with the existing literature. Research on ChatGPT in medical education primarily focuses on “medical knowledge,” “educational assessment,” “clinical decision-making,” and “exam performance” [50,71,72], with increasing attention to ethics concerns [73]. Previous studies have documented the increase in publications in the field, as well as the central role played by the United States [74]. Analyzing collaboration networks and journals may provide researchers in this field with a comprehensive understanding of institutional collaboration and journal publication information. Moreover, through thematic analysis, the evolution of research topics and recent research hot spots in this field were revealed.

### Implications of the Findings

The findings of this study have a number of implications. The considerable increase in research on ChatGPT in medicine indicates its extensive integration into medical education and clinical practice. The strong international collaboration suggests that research outcomes related to ChatGPT in medical education are being shared worldwide. Authors conduct research in multiple small and isolated groups. This means that researchers in this field tend to work in independent teams and lack communication across different teams. The results of the dual-map overlay of the journals showed that both the citing and cited journals were from the field of medicine, indicating that research on ChatGPT in medical education has not yet been integrated with other disciplines, failing to form a cross-disciplinary trend.

Research themes evolved from preclinical education to clinical practice simulation, confirmed via keyword and citation analysis. As a learning tool, ChatGPT has passed medical licensing exams in the United States [75,76], India [77], the United Kingdom [78], and South Korea [79]. Its applications have expanded from basic exams to specialty tests, including the American Orthopaedic In-Training Examination [80], the Membership of the Royal Colleges of Physicians of the United Kingdom exam [81], and the Chinese Critical Care Examination [82]. Researchers test ChatGPT’s medical knowledge through multiple exams. Nowadays, assessing the feasibility of ChatGPT as a learning tool is a research hot spot. Concurrently with the release of LLMs such as Microsoft Copilot, Google Gemini, and China’s DeepSeek, studies have begun to compare different models’ exam performance [83-85]. However, as LLMs keep evolving, we urgently need rigorous evidence of their reliability in medical testing. This proof remains essential before medical students fully adopt these learning tools.

In clinical teaching, ChatGPT has been used to emulate a range of clinical scenarios, including undiagnosed diabetes, kidney injury, and ophthalmic diseases [18-20]. This creates an interactive clinical reasoning environment for students, enhancing engagement during learning. However, it is important to note that ChatGPT sometimes generates inaccurate or fabricated information [86,87]. Medical educators and students need a clear understanding of ChatGPT’s capabilities and limitations across medical specialties to effectively use AI tools for teaching and learning.

ChatGPT’s integration into medical education raises ethical issues, highlighted by our keyword analysis. Researchers are concerned that it could unintentionally reveal a patient’s personal information [88]. However, little research has been conducted on ChatGPT in medical ethics education (eg, medical ethics courses) and its educational impact [89]. While many studies have evaluated ChatGPT’s performance in medical licensing exams across various countries, research on its ability to address medical ethics issues remains limited. The 2024 study by Danehy et al [90] showed that GPT-3.5 and ChatGPT-4 performed worse on ethics questions than on medical knowledge questions. This suggests that ChatGPT’s training emphasizes medical knowledge over medical ethics. This training bias may be a potential trigger for ethical controversies involving ChatGPT in clinical practice.

### Study Strengths and Limitations

This study has both strengths and limitations. To our knowledge, this is the first study to use bibliometric analysis to study the use of ChatGPT in medical education rather than general medicine. Furthermore, the visualization of quantitative results provides a comprehensive understanding of the current status of publications, research hot spots, and development trends related to ChatGPT in medical education.

Despite best efforts to include all the relevant terms and terminology in the literature search, some relevant papers may have been omitted. The search was confined to Web of Science, and only research articles written in English were included, with articles in other languages not being considered. In addition, due to the ongoing nature of the research, recent high-quality studies may not have been included.

Subsequently, the discussion focus on providing strong evidence to demonstrate the feasibility of ChatGPT as a learning tool, evaluating ChatGPT’s medical ethics awareness in medical education, and offering evidence to support the application of ChatGPT in medical ethics.

### Conclusions

In conclusion, this bibliometric analysis of ChatGPT in medical education reveals characteristics such as rapid publication growth, concentrated contributions from leading countries and institutions, decentralized author networks, and evolving thematic focuses. It will be crucial to enhance institution collaboration and cross-team partnerships in the future. This will promote the application potential of ChatGPT in various fields of medical education. Improving the effectiveness of ChatGPT is expected to provide educators and students with a more efficient medical teaching and learning process.

## Abbreviations

AI: artificial intelligence
BC: betweenness centrality
IF: impact factor
LLM: large language model
RQ: research question
USMLE: United States Medical Licensing Examination
